# Smoking history and breast cancer risk by pathological subtype: MCC-Spain study

**DOI:** 10.18332/tid/174132

**Published:** 2023-11-30

**Authors:** Belén Peñalver-Argüeso, Esther García-Esquinas, Adela Castelló, Nerea Fernández de Larrea-Baz, Gemma Castaño-Vinyals, Pilar Amiano, Tania Fernández-Villa, Marcela Guevara, Guillermo Fernández-Tardón, Juan Alguacil, Mireia Obón-Santacana, Inés Gómez-Acebo, Marina Pinto-Carbó, Rafael Marcos-Gragera, Nuria Aragonés, Amaia Aizpurua, Vicente Martín-Sánchez, Eva Ardanaz, Trinidad Dierssen-Sotos, Jose Juan Jiménez-Moleón, Manolis Kogevinas, Marina Pollán, Beatriz Pérez-Gómez

**Affiliations:** 1 Public Health and Preventive Medicine Teaching Unit, National School of Public Health, Carlos III Institute of Health, Madrid, Spain; 2 Department of Epidemiology of Chronic Diseases, National Centre for Epidemiology, Carlos III Institute of Health, Madrid, Spain; 3 Department of Preventive Medicine, Public Health and Microbiology, School of Medicine, Autonomous University of Madrid, Madrid, Spain; 4 Consortium for Biomedical Research in Epidemiology and Public Health, Madrid, Spain; 5 Barcelona Institute for Global Health, Barcelona, Spain; 6 Department of Medicine and Life Sciences, Universitat Pompeu Fabra, Barcelona, Spain; 7 Hospital del Mar Medical Research Institute, Barcelona, Spain; 8 Ministry of Health of the Basque Government, Sub Directorate for Public Health and Addictions of Gipuzkoa, San Sebastián, Spain; 9 Epidemiology of Chronic and Communicable Diseases Group, Biodonostia Health Research Institute, San Sebastián, Spain; 10 Research Group on Gene-Environment Interactions and Health, Institute of Biomedicine, Universidad de León, León, Spain; 11 Public Health Institute of Navarra, Pamplona, Spain; 12 Navarra Institute for Health Research, Pamplona, Spain; 13 Health Research Institute of Asturias, Oviedo, Spain; 14 University Institute of Oncology of Asturias, Oviedo, Spain; 15 Natural Resources, Health and Environment Research Center, University of Huelva, Huelva, Spain; 16 Biomarkers and Susceptibility Unit, Oncology Data Analytics Program, Catalan Institute of Oncology, Barcelona, Spain; 17 ONCOBELL Program, Bellvitge Biomedical Research Institute, L'Hospitalet de Llobregat, Barcelona, Spain; 18 Instituto de Investigación Marqués de Valdecilla, Santander, Spain; 19 Faculty of Medicine, University of Cantabria, Santander, Spain; 20 Cancer and Public Health Research Area, Foundation for the Promotion of Health and Biomedical Research, Valencia, Spain; 21 Epidemiology Unit and Girona Cancer Registry, Oncology Coordination Plan, Department of Health, Autonomous Government of Catalonia, Catalan Institute of Oncology, Girona, Spain; 22 Descriptive Epidemiology, Genetics and Cancer Prevention Group, Girona Biomedical Research Institute, Girona, Spain; 23 Epidemiology Section, Public Health Division, Department of Health of Madrid, Madrid, Spain; 24 Instituto de Investigación Biosanitaria de Granada, Hospitales Universitarios de Granada, Universidad de Granada, Granada, Spain

**Keywords:** breast cancer, smoking, obesity, hormone receptor, HER2

## Abstract

**INTRODUCTION:**

The role of cigarette smoking on breast cancer risk remains controversial, due to its dual carcinogenic-antiestrogenic action.

**METHODS:**

In the population-based multi-case-control study (MCC-Spain), we collected epidemiological and clinical information for 1733 breast cancer cases and 1903 controls, including smoking exposure. The association with breast cancer, overall, by pathological subtype and menopausal status, was assessed using logistic and multinomial regression models.

**RESULTS:**

Smokers had higher risk of premenopausal breast cancer, particularly if they had smoked ≥30 years (AOR=1.75; 95% CI: 1.04–2.94), although most estimates did not achieve statistical significance. In contrast, among postmenopausal women, smoking was associated with lower risk of breast cancer, mainly in overweight and obese women. The strongest risk reductions were observed among postmenopausal women who had stopped smoking ≥10 years before cancer diagnosis, particularly for HER2+ tumors (AOR=0.28; 95% CI: 0.11–0.68); p for heterogeneity = 0.040). Also, those who had smoked <10 pack-years (AOR=0.68; 95% CI: 0.47–0.98) or 10–25 pack-years (AOR=0.62; 95% CI: 0.42–0.92) during their lifetime were at a reduced risk of all breast cancer subtypes (p for heterogeneity: 0.405 and 0.475, respectively); however, women who had smoked more than 25 pack-years showed no reduced risk.

**CONCLUSIONS:**

Menopausal status plays a key role in the relationship between tobacco and breast cancer for all cancer subtypes. While smoking seems to increase the risk in premenopausal woman, it might be associated to lower risk of breast cancer among postmenopausal women with excess weight.

## INTRODUCTION

Despite advances in diagnostic methods and treatments, breast cancer remains currently the most common cancer in women and a major cause of disease burden across the world^1^. During the last decades, a number of epidemiological studies have evaluated the effects of tobacco smoking on breast cancer, but this relationship remains unclear. While some studies have found an increased risk among women who have smoked heavily or during long periods^2,3^, others suggest this association may be limited to women who have smoked between puberty and the first full-term pregnancy^4,5^, and others fail to observe any increased risk^6,7^. Difficulties disentangling this association possibly derive from its complexity. On the one hand, the timing of exposure may play a relevant role due to the breastrelated windows of susceptibility^8^; on the other hand, smoking combines the presence of carcinogens – which have been shown to induce mammary tumors in rodents^9,10^ – with the antiestrogenic properties of tobacco^11^.

Additionally, due to the heightened relevance of adipose tissue as estrogenic producer after menopause, it has been proposed that obesity may modify this relationship^12,13^. Further, some studies suggest that the effect of smoking might differ by pathological subtype, especially considering that the differences between prognostic as well as molecular and cellular characteristics seem to indicate unique etiologies for each subtype^14,15^.

In spite of proposed hypotheses explaining the effects of tobacco on the breast, controversies over cancer subtypes and the importance of the dual carcinogenic-antiestrogenic effect continue to arise. In this study, we examine the relationship between active cigarette smoking and invasive breast cancer risk, focusing on the differences according to menopausal status, body mass index (BMI) and tumor subtype.

## METHODS

### Study population

The multi-case-control (MCC-Spain) study is a population-based case-control study conducted between 2008 and 2013 in 12 Spanish provinces to identify environmental, lifestyle and genetic factors related to 5 tumors: breast, prostate, colorectal, gastric, and chronic lymphocytic leukemia. It used a single set of population-based controls, frequency matched by age and sex, with the overall distribution of cases for each province. The study design has been described elsewhere^16^.

The cases were recruited in 22 collaborating hospitals and identified as soon as possible after diagnosis through active search and periodical visits to the collaborating hospital departments. Population-based controls, randomly selected from the general practitioner lists of the catchment area of each collaborating hospital, were invited to participate by phone^17^. Participants had to be aged 20–85 years, to have lived within the recruitment area at least 6 months prior to inclusion in the study, to be able to answer the epidemiological questionnaire, and not to have had a previous history of the disease, breast cancer in this case. The Ethics Committee of the participant institutions approved the study protocol. All participants were informed about the study objectives and provided written informed consent. For the present study, a subset of 1733 histologically confirmed incident breast cancer cases and 1903 population controls was included (Supplementary file Figure 1). Response rates were 69% for breast cancer cases and 63% for controls. Cases were classified according to pathological subtype into three groups: 1) Estrogen or progesterone receptor positive (ER/PR) without overexpression of the human epidermal growth factor receptor 2 (HER2) (HR+); 2) HER2 positive (HER2+), and 3) triple-negative (TN), i.e. ER-, PR- and HER2-.

### Data collection

MCC-Spain participants were administered an epidemiological questionnaire by trained personnel in a face-to-face interview, filled a food frequency questionnaire and donated biological samples. The epidemiological questionnaire registered sociodemographic and self-reported anthropometric data (height and weight one year before the interview), and collected information on personal and family history of cancer, pre-existing medical conditions, residential and occupational history, tobacco use and physical activity previous to diagnosis. Detailed information was also collected on gynecological and obstetric variables including age at menarche, parity, age at first birth, menopausal status, age at menopause, history of benign breast disease, family history of breast cancer, oral contraceptive and postmenopausal hormone therapy use.

Tobacco smoking variables including age at initiation, duration and intensity were truncated to take into account a minimum latency period of one year before cancer diagnosis. Women who reported having smoked <100 cigarettes during their lifetime were classified as never smokers. Former smokers were classified according to the time since cessation (<10 or ≥10 years). Additionally, former and current smokers were classified according to the age at smoking initiation, duration (<20, 20–30, >30 years), and intensity of consumption (<15, or ≥15 cigarettes/ day). Duration and intensity of tobacco consumption were combined to generate a measurement variable for cumulative exposure (<10, 10–25, >25 pack-years). Finally, taking into account the information on reproductive history, parous women were classified according to the time (<10 or ≥10 years) and the number of cigarettes (<15 or ≥15 cigarettes/day) they smoked before their first birth.

### Statistical analysis

Sociodemographic and smoking characteristics are described with mean and standard deviation values (numerical variables), or frequencies and percentages (categorical variables). Differences between cases and controls were assessed using Student’s t-test and chi-squared test, respectively, assuming equal variances. The association between breast cancer risk and each smoking variable was examined using unconditional logistic regression models which included the matching variables (age at recruitment and province of residence) and education level, as cases and controls were unbalanced for this factor. Multivariable models were further adjusted for known breast cancer risk factors: age at first birth, number of children, menopausal status, previous breast biopsies, family history of breast cancer, and alcohol consumption.

Stratified analyses were conducted by menopausal status (pre-/peri-menopausal; post-menopausal) and by body mass index (BMI, kg/m^2^) in post-menopausal women, and effect modification was tested with likelihood ratio tests that compared models with and without interaction terms.

Models for pre- and post-menopausal women were additionally adjusted for oral contraceptive use in the case of pre-menopausal women, and for BMI, hormone replacement therapy and age at menopause in post-menopausal women. Differences in the studied associations according to breast cancer subtypes were explored with multinomial logistic regression models that adjusted for the variables described above. Furthermore, we tested for heterogeneity of effects of each tobacco variable between tumor subtypes.

In addition, we performed sensitivity analyses including diet-related variables (dietary pattern, daily caloric intake and antioxidant activity) in the adjusting models. Results are expressed as adjusted odds ratio (AOR) or relative risk ratio (RRR), with 95% confidence interval (95% CI). All analyses were performed with the statistical package Stata 15/IC (Stata Corp. College Station, TX, USA).

### Ethics

The MCC-Spain study was approved by the Ethics Committee of each collaborating institution, in conformity with the principles of the Declaration of Helsinki. Participants were informed about the objectives and signed an informed consent. Personal identifiers are removed from the datasets in order to secure the confidentiality of the subjects. Researchers were asked to sign a confidentiality agreement. The database was recorded in the Spanish Agency for Data Protection (Number: 2102672171).

## RESULTS

### Sample description

A total of 1733 breast cancer cases and 1903 controls were included in the analysis. Table 1 presents the main characteristics of the sample. Cases were slightly younger than controls, had fewer children, were more likely to be pre- or peri-menopausal, and had more frequently family history of breast cancer. The prevalence of obesity was higher among pre-menopausal controls and post-menopausal breast cancer cases than among their counterparts. In addition, the prevalence and intensity of smoking was higher among breast cancer cases.

**Table 1:** Distribution of sociodemographic and smoking characteristics in breast cancer cases and controls, multi-case-control study (MCC-Spain), 2008–2013, Spain (N=3636)

*Characteristics*	*Total*			*Pre-menopausal*			*Post-menopausal*		
	*Cases n (%)*	*Controls n (%)*	*p*	*Cases n (%)*	*Controls n (%)*	*p*	*Cases n (%)*	*Controls n (%)*	*p*
	*(N=1733)*	*(N=1903)*		*(N=610)*	*(N=547)*		*(N=1122)*	*(N=1352)*	
***Sociodemographic***									
**Age** (years), mean (SD)	56.4 (12.6)	59.1 (13.2)	<0.001	44.0 (6.4)	43.5 (6.1)	0.154	63.1 (9.7)	65.4 (9.6)	<0.001
**Education level**									
No education/primary studies	831 (48.0)	912 (47.9)		149 (24.4)	109 (19.9)		682 (60.8)	801 (59.2)	
Secondary school	573 (33.1)	588 (30.9)		282 (46.2)	228 (41.7)		290 (25.8)	359 (26.6)	
University graduate	329 (19.0)	403 (21.2)	0.174	179 (29.3)	210 (38.4)	0.004	150 (13.4)	192 (14.2)	0.717
**Body mass index** (kg/m²)									
<18.5	28 (1.8)	37 (2.3)		18 (3.2)	21 (4.3)		10 (1.0)	16 (1.4)	
18.5–25.0	741 (46.3)	809 (49.3)		368 (65.8)	299 (60.9)		372 (35.8)	510 (44.3)	
25.0–30.0	541 (33.8)	509 (31.0)		135 (24.2)	113 (23.0)		406 (39.0)	396 (34.4)	
≥30.0	290 (18.1)	287 (17.5)	0.197	38 (6.8)	58 (11.8)	0.028	252 (24.2)	229 (19.9)	<0.001
**Alcohol intake** (g/day)									
Non-drinker	530 (34.1)	649 (30.6)		171 (28.0)	156 (28.5)		359 (32.0)	491 (36.3)	
<7	544 (30.1)	572 (31.4)		242 (39.7)	203 (37.1)		302 (26.9)	369 (27.3)	
≥7	439 (24.1)	458 (25.3)		130 (21.3)	125 (22.9)		308 (27.5)	333 (24.6)	
Amount unknown	224 (11.8)	220 (12.7)	0.106	67 (11.0)	63 (11.5)	0.665	153 (13.6)	159 (11.8)	0.082
**Age at menarche,** mean (SD)	12.8 (1.6)	12.9 (1.6)	0.120	12.6 (1.4)	12.7 (1.5)	0.602	12.8 (1.6)	12.9 (1.7)	0.211
**Age at first birth,** mean (SD)	26.7 (5.0)	26.5 (4.8)	0.270	28.0 (5.6)	28.0 (5.6)	0.986	26.1 (4.5)	26.0 (4.3)	0.743
**Number of children**									
None	351 (20.3)	355 (18.7)		155 (25.5)	153 (28.2)		196 (17.5)	202 (15.0)	
1–2	993 (57.5)	1042 (55.0)		392 (64.4)	340 (62.6)		601 (53.8)	700 (51.9)	
3–4	336 (19.5)	406 (21.4)		58 (9.5)	44 (8.1)		278 (24.9)	361 (26.8)	
>4	47 (2.7)	92 (4.9)	0.002	4 (0.7)	6 (1.1)	0.521	43 (3.8)	86 (6.4)	0.011
**Oral contraceptive use** (years)									
Never	914 (52.9)	971 (51.1)		211 (34.7)	163 (29.9)		703 (62.8)	808 (59.9)	
≤5	370 (21.4)	375 (19.7)		162 (26.6)	149 (27.3)		208 (18.6)	224 (16.6)	
>5	216 (12.5)	284 (15.0)		119 (19.6)	127 (23.3)		97 (8.7)	156 (11.6)	
Duration unknown	227 (13.1)	269 (14.2)	0.060	116 (19.1)	107 (19.6)	0.138	111 (9.9)	162 (12.0)	0.031
**Age at menopause,** mean (SD)	**-**	**-**	**-**	**-**	**-**	**-**	49.1 (5.3)	48.4 (5.3)	0.005
**Hormone replacement therapy** (years)									
Never	-	-	-	-	-	-	965 (89.3)	1147 (89.4)	
≤5	-	-	-	-	-	-	74 (6.8)	89 (6.9)	
>5	-	-	-	-	-	-	31 (2.9)	37 (2.9)	
Duration unknown	-	-	-	-	-	-	11 (1.0)	10 (0.8)	0.997
**Previous biopsies**									
None	1571 (92.5)	1797 (98.0)		566 (94.8)	534 (98.2)		1005 (91.3)	1263 (97.9)	
Yes	127 (7.5)	37 (2.0)	<0.001	31 (5.2)	10 (1.8)	0.002	96 (8.7)	27 (2.1)	<0.001
**Family history of breast cancer**									
None	1303 (75.2)	1631 (85.7)		432 (70.8)	470 (85.9)		870 (77.5)	1157 (85.6)	
Second degree only	174 (10.0)	106 (5.6)		88 (14.4)	48 (8.8)		86 (7.7)	58 (4.3)	
1 First degree	224 (12.9)	155 (8.1)		81 (13.3)	29 (5.3)		143 (12.7)	126 (9.3)	
First degree	32 (1.8)	11 (0.6)	<0.001	9 (1.5)	0 (0.0)	<0.001	23 (2.0)	11 (0.8)	<0.001
***Smoking***									
**Smoking status 1 year before the interview**									
Never smoker	972 (56.4)	1142 (60.1)		242 (40.0)	237 (43.5)		730 (65.4)	904 (66.9)	
Former smoker ≥10	170 (9.9)	180 (9.5)		61 (10.1)	53 (9.7)		109 (9.8)	126 (9.3)	
Former smoker <10	168 (9.8)	189 (9.9)		83 (13.7)	80 (14.7)		85 (7.6)	109 (8.1)	
Active smoker	413 (24.0)	390 (20.5)	0.068	219 (36.2)	175 (32.1)	0.483	193 (17.3)	213 (15.8)	0.715
**Duration of tobacco use** (years)									
Never smoker	972 (56.5)	1142 (60.2)		242 (40.1)	237 (43.5)		730 (65.5)	904 (67.0)	
<20	247 (14.4)	229 (12.1)		151 (25.0)	130 (23.9)		96 (8.6)	98 (7.3)	
20–30	249 (14.5)	228 (12.0)		150 (24.8)	138 (25.3)		99 (8.9)	89 (6.6)	
>30	251 (14.6)	299 (15.8)	0.014	61 (10.1)	40 (7.3)	0.325	189 (17.0)	258 (19.1)	0.061
**Intensity** (cigarettes/day)									
Never smoker	972 (56.8)	1142 (60.9)		242 (40.2)	237 (44.0)		730 (65.8)	904 (67.8)	
<15	356 (20.8)	363 (19.3)		180 (29.9)	153 (28.4)		175 (15.8)	209 (15.7)	
≥15	384 (22.4)	371 (19.8)	0.039	180 (29.9)	149 (27.6)	0.430	204 (18.4)	220 (16.5)	0.447
**Years smoking before first birth**									
Never smoker	801 (61.2)	939 (64.7)		177 (40.0)	162 (42.5)		624 (72.1)	776 (72.7)	
<10	255 (19.5)	253 (17.4)		118 (26.6)	87 (22.8)		137 (15.8)	165 (15.4)	
≥10	253 (19.3)	260 (17.9)	0.161	148 (33.4)	132 (34.6)	0.447	105 (12.1)	127 (11.9)	0.957
**Cigarettes per day before first birth**									
Never smoker	801 (65.8)	939 (68.2)		177 (44.3)	162 (47.0)		-801	776 (75.4)	
<15	211 (17.3)	226 (16.4)		117 (29.3)	95 (27.5)		94 (11.5)	130 (12.6)	
≥15	206 (16.9)	212 (15.4)	0.404	106 (26.5)	88 (25.5)	0.756	100 (12.2)	123 (12.0)	0.754

Chi-squared test or Student’s t-test were used to assess differences between cases and controls for each variable.

### Tobacco variables and breast cancer risk

**Figure 1:**
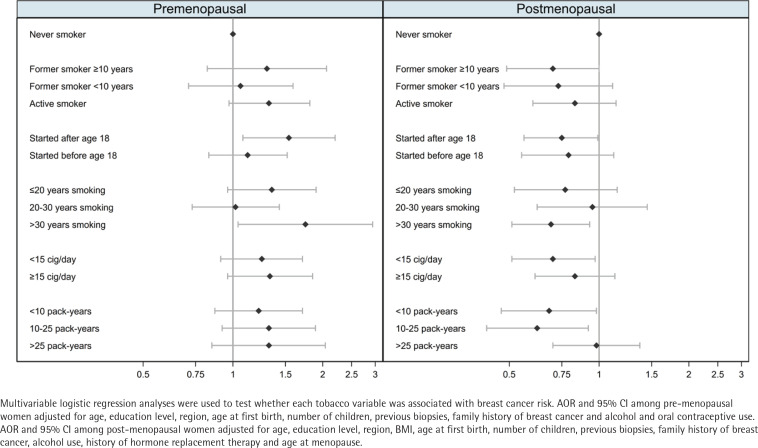
Adjusted odds ratio for the risk of female breast cancer associated with tobacco use by menopausal status, multi-case-control study (MCC-Spain), 2008–2013, Spain (Pre-menopausal women: N=1157; Cases=610; Controls=547; Post-menopausal women: N=2474; Cases=1122; Controls=1352)

Table 2 shows the results for the association between measures of smoking exposure and breast cancer risk, with clear differences observed by menopausal status (Figure 1). Among pre-menopausal women, tobacco exposure was associated with an increased risk of breast cancer, with the strongest effects observed for women who had started smoking after the age of 18 years (AOR=1.54; 95% CI: 1.08–2.20), or who had smoked for >30 years (AOR=1.75; 95% CI: 1.04–2.94). In these women, risk estimators for intensity and cumulative exposure pointed towards an excess of risk, while smoking before the first birth showed no effect on breast cancer. Among post-menopausal participants, smoking was associated with a decreased risk of breast cancer. Of note, we observed a lower risk of breast cancer with long-term maintained smoking of >30 years (AOR=0.69; 95% CI: 0.51–0.93) and in women with a low intensity of smoking of <15 cigarettes/day (AOR=0.70; 95% CI: 0.51–0.97). Combining these two variables into pack-years, women who had smoked less than 10 packyears or 10–25 pack-years during their lifetime were at a reduced risk of post-menopausal breast cancer: <10 pack-years (AOR=0.68; 95% CI: 0.47–0.98); and 10–25 pack-years (AOR=0.62; 95% CI: 0.42–0.92). However, those who had smoked >25 pack-years showed no decreased risk. Smoking before the first birth was also associated with a reduced risk of postmenopausal breast cancer. The sensitivity analysis (Supplementary file Table S1) yielded no differences with these results.

**Table:** Table 2. Association between cigarette smoking variables and breast cancer risk, overall and by menopausal status, multi-case-control study (MCC-Spain), 2008–2013, Spain (N=3636)

*Smoking variables*	*All women (N=3636; Cases=1733; Controls=1903)*						*Pre-menopausal women (N=1157; Cases=610; Controls=547)*						*Post-menopausal women (N=2474; Cases=1122; Controls=1352)*						*p-int^f^*
	*Ca*	*Co*	*AOR ^b^*	*95% CI*	*AOR ^c^*	*95% CI*	*Ca*	*Co*	*AOR^b^*	*95% CI*	*AOR ^d^*	*95% CI*	*Ca*	*Co*	*AOR ^b^*	*95% CI*	*AOR^e^*	*95% CI*	
Smoking status (1 year before the interview)																			
Never smoker	972	1142	1.00		1.00		242	237	1.00		1.00		730	904	1.00		1.00		
Former smoker ≥10	170	180	1.12	0.88–1.41	0.92	0.71–1.20	61	53	1.16	0.76–1.77	1.30	0.82–2.06	109	126	1.01	0.76–1.35	0.70	0.49–1.00	
Former smoker <10	168	189	0.90	0.71–1.15	0.87	0.66–1.14	83	80	1.01	0.70–1.45	1.06	0.71–1.59	85	109	0.78	0.57–1.07	0.73	0.48–1.11	
Active smoker	413	390	1.03	0.87–1.24	1.02	0.83–1.26	219	175	1.19	0.91–1.57	1.32	0.97–1.81	193	213	0.87	0.68–1.11	0.83	0.60–1.14	0.103
p–trend				0.892		0.972				0.259		0.107				0.162		0.143	
**Age at smoking initiation**																			
Never smoker	972	1142	1.00		1.00		242	237	1.00		1.00		730	904	1.00		1.00		
≥18	367	369	1.09	0.91–1.30	0.97	0.79–1.19	140	101	1.33	0.97–1.83	1.54	1.08–2.20	227	267	0.93	0.75–1.16	0.75	0.56–0.99	
<18	389	389	0.97	0.81–1.17	0.95	0.77–1.18	227	209	1.05	0.80–1.37	1.12	0.83–1.52	161	178	0.85	0.65–1.11	0.79	0.55–1.12	0.011
p–trend				0.955		0.649				0.686		0.414				0.219		0.073	
**Duration of tobacco use** (years)																			
Never smoker	972	1142	1.00		1.00		242	237	1.00		1.00		730	904	1.00		1.00		
≤20	247	229	1.08	0.87–1.34	1.05	0.82–1.34	151	130	1.17	0.87–1.50	1.35	0.96–1.90	96	98	1.05	0.76–1.44	0.77	0.52–1.15	
20–30	249	228	1.07	0.86–1.32	0.99	0.77–1.26	150	138	1.01	0.75–1.37	1.02	0.73–1.43	99	89	1.09	0.78–1.51	0.95	0.62–1.45	
>30	251	299	0.96	0.79–1.16	0.87	0.69–1.09	61	40	1.45	0.91–2.30	1.75	1.04–2.94	189	258	0.77	0.61–0.97	0.69	0.51–0.93	0.001
p–trend				0.863		0.272				0.284		0.138				0.052		0.025	
**Intensity** (cigarettes/day)																			
Never smoker	972	1142	1.00		1.00		242	237	1.00		1.00		730	904	1.00		1.00		
<15	356	363	1.00	0.84–1.20	0.96	0.78–1.18	180	153	1.13	0.85–1.51	1.25	0.91–1.71	175	209	0.85	0.67–1.09	0.70	0.51–0.97	
≥15	384	371	1.09	0.91–1.30	0.99	0.80–1.22	180	149	1.18	0.88–1.58	1.33	0.96–1.85	204	220	0.97	0.77–1.23	0.83	0.61–1.13	0.050
p–trend				0.378		0.876				0.244		0.075				0.655		0.129	
**Cumulative exposure** (pack-years)																			
Never smoker	972	1142	1.00		1.00		242	237	1.00		1.00		730	904	1.00		1.00		
<10	254	256	0.98	0.80-1.21	0.93	0.74-1.18	140	127	1.08	0.79-1.47	1.22	0.87-1.71	113	128	0.89	0.66-1.19	0.68	0.47-0.98	
10-25	228	240	0.97	0.78-1.20	0.90	0.71-1.15	133	105	1.23	0.90-1.69	1.32	0.92-1.89	95	134	0.74	0.55-1.00	0.62	0.42-0.92	
>25	247	234	1.16	0.94-1.42	1.06	0.83-1.35	82	68	1.15	0.78-1.68	1.32	0.85-2.04	165	165	1.05	0.81-1.36	0.98	0.70-1.37	0.059
p-trend				0.297		0.952				0.248		0.097				0.733		0.343	
**Years smoking before first birth^a^**																			
Never smoker	801	939	1.00		1.00		177	162	1.00		1.00		624	776	1.00		1.00		
<10	255	253	0.99	0.80-1.23	0.94	0.73-1.21	118	87	1.18	0.82-1.70	1.35	0.88-2.07	137	165	0.85	0.64-1.12	0.65	0.45-0.94	
≥10	253	260	0.97	0.78-1.21	0.96	0.74-1.24	148	132	1.06	0.77-1.47	1.11	0.75-1.64	105	127	0.87	0.63-1.19	0.83	0.54-1.26	0.034
p-trend				0.789		0.675				0.686		0.466				0.267		0.137	
**Cigarettes per day before first birth^a^**																			
Never smoker	801	939	1.00		1.00		177	162	1.00		1.00		624	776	1.00		1.00		
<15	211	226	0.92	0.73-1.16	0.91	0.70-1.18	117	95	1.09	0.77-1.56	1.27	0.85-1.90	94	130	0.77	0.56-1.05	0.60	0.40-0.90	
≥15	206	212	0.98	0.78-1.24	0.96	0.73-1.25	106	88	1.15	0.80-1.66	1.23	0.81-1.87	100	123	0.86	0.63-1.18	0.83	0.55-1.27	0.053
p-trend				0.775		0.639				0.441		0.288				0.208		0.147	

AOR: adjusted odds ratio. Multivariable logistic regression analyses were used to test whether each tobacco variable was associated with breast cancer risk. The p-trend values were calculated by incorporating the categorized variable as a continuous variable in the multivariable models. The p-value of the interaction term between menopausal status and the corresponding variable was calculated using the likelihood ratio test.

a Only parous women.

b OR and 95% CI adjusted for age, education level and region.

c OR and 95% CI adjusted for age, education level, region, BMI, age at first birth, number of children, previous biopsies, family history of breast cancer, alcohol use and menopausal status.

d OR and 95% CI adjusted for age, education level, region, age at first birth, number of children, previous biopsies, family history of breast cancer and alcohol and oral contraceptive use.

e OR and 95% CI adjusted for age, education level, region, BMI, age at first birth, number of children, previous biopsies, family history of breast cancer, alcohol use, history of hormone replacement therapy and age at menopause.

f p-int.: p-value of the interaction term.

Results of post-menopausal women stratified by BMI (under/normal weight vs overweight/ obese) found that the protective effects of tobacco were restricted to overweight and obese women (Table 3). Among non-obese women, no association was found between smoking exposure and breast cancer risk, while the duration and intensity of exposure showed a significant dose-response effect among women with BMI >25.0 kg/m^2^.

**Table:** Table 3. Association between cigarette smoking variables and breast cancer risk in post-menopausal nonobese and obese women, multi-case-control study (MCC-Spain), 2008–2013, Spain (N=2191)

*Smoking variables*	*Under/normal weight (N=908)*				*Overweight/obese (N=1283)*				*p-int ^c^*
	*Ca*	*Co*	*AOR ^b^*	*95% CI*	*Ca*	*Co*	*AOR ^b^*	*95% CI*	
**Smoking status** (1 year before the interview)									
Never smoker	214	305	1.00		447	430	1.00		
Former ≥10	46	55	1.00	0.57–1.75	58	65	0.55	0.34–0.90	
Former <10	37	52	0.99	0.53–1.86	47	49	0.64	0.35–1.16	
Active smoker	85	114	0.89	0.55–1.45	101	81	0.77	0.49–1.19	0.534
p-trend				0.663				0.107	
**Age at smoking initiation** (years)									
Never smoker	214	305	1.00		447	430	1.00		
≥18	86	128	0.89	0.57–1.38	131	124	0.67	0.46–0.97	
<18	80	92	1.01	0.60–1.70	78	70	0.66	0.40–1.09	0.554
p-trend				0.932				0.031	
**Duration of tobacco use** (years)									
Never smoker	214	305	1.00		447	430	1.00		
<20	40	40	1.02	0.54–1.93	51	52	0.67	0.40–1.12	
20–30	43	42	1.57	0.83–2.96	54	44	0.64	0.36–1.15	
>30	83	138	0.75	0.47–1.18	100	98	0.68	0.44–1.03	0.281
p-trend				0.360				0.036	
**Intensity** (cigarettes/day)									
Never smoker	214	305	1.00		447	430	1.00		
<15	82	105	0.88	0.55–1.42	85	87	0.59	0.39–0.91	
≥15	81	105	1.05	0.65–1.70	118	101	0.74	0.49–1.13	0.427
p-trend				0.917				0.065	
**Cumulative exposure** (pack-years)									
Never smoker	214	305	1.00		447	430	1.00		
<10	55	63	0.90	0.51–1.58	52	58	0.56	0.34–0.92	
10–25	40	65	0.77	0.42–1.40	54	58	0.53	0.31–0.92	
>25	67	82	1.14	0.69–1.90	92	71	0.92	0.58–1.48	0.611
p-trend				0.836				0.202	
**Years smoking before first birth^a^**									
Never smoker	168	250	1.00		397	379	1.00		
<10	63	82	0.81	0.46–1.41	73	70	0.56	0.33–0.95	
≥10	49	70	0.98	0.51–1.87	52	50	0.76	0.43–1.34	0.533
p-trend				0.773				0.131	
**Cigarettes per day before first birth^a^**									
Never smoker	168	250	1.00		397	379	1.00		
<15	42	69	0.74	0.40–1.37	50	52	0.54	0.31–0.96	
≥15	44	65	1.01	0.55–1.87	54	50	0.73	0.40–1.35	0.695
p-trend				0.849				0.118	

AOR: adjusted odds ratio. Multivariable logistic regression analyses were used to test whether each tobacco variable was associated with breast cancer risk. The p-trend values were calculated by incorporating the categorized variable as a continuous variable in the multivariable models. The p-value of the interaction term between weight status and the corresponding variable was calculated using the likelihood ratio test.

a Only parous women.

b AOR and 95% CI adjusted for age, education level, region, previous biopsies, family history of breast cancer, age at first birth, number of children, alcohol use, history of hormone replacement therapy and age at menopause.

c p-int.: p-value of the interaction term.

### Tobacco variables and tumor subtypes

Pathological tumor subtype information was available for 1578 (91.1%) cases. Only 300 cases were HER2+ and 134 were TN, limiting our statistical power to detect differences among these subgroups. Table 4 shows the results for the association between measures of smoking exposure and breast cancer pathological subtypes. Among pre-menopausal women, results were consistent across subtypes. In post-menopausal women, former smokers who had stopped smoking ≥10 years before cancer diagnosis showed the strongest risk reductions, particularly for HER2+ tumors: (RRR=0.28; 95% CI: 0.11–0.68; p heterogeneity across subtypes = 0.040). In regard to duration and intensity of tobacco consumption, the results were consistent across subtypes, although it is interesting to highlight a very strong risk reduction of TN tumors observed in post-menopausal women who smoked <15 cigarettes/day (RRR=0.28; 95% CI: 0.08–0.97). Protective benefits of having smoked before the first birth showed the strongest reductions for HER2+ tumors, although, again, no significant heterogeneity across subtypes was observed. The sensitivity analysis (Supplementary file Table S2) showed some differences, although there were no substantial alterations regarding the sign of the association or the risk-related variables.

**Table 4:** Association between cigarette smoking variables and breast cancer subtypes, overall and by menopausal status, multi-case-control study (MCC-Spain), 2008–2013, Spain (N=3636)

*Smoking variables*	*All Women (N=3636; Cases=1733; Controls=1903)*											*Pre-menopausal Women (N=1157; Cases=610; Controls=547)*											*Post-menopausal Women (N=2474; Cases=1122; Controls=1352)*										
	*Co*	*HR+ (N= 1144)*			*HER2+ (N =300)*			*TN (N= 134)*				*Co*	*HR+ (N= 405)*			*HER2+ (N = 105)*			*TN (N= 49)*				*Co*	*HR+ (N= 738)*			*HER2+ (N = 195)*			*TN (N= 85)*			
	*n*	*n*	*RRR^a^*	*95% CI*	*n*	*RRR^a^*	*95% CI*	*n*	*RRR^a^*	*95% CI*	*p-het*	*n*	*n*	*RRR^b^*	*95% CI*	*n*	*RRR^b^*	*95% CI*	*n*	*RRR^b^*	*95% CI*	*p-het*	*n*	*n*	*RRR^c^*	*95% CI*	*n*	*RRR^c^*	*95% CI*	*n*	*RRR^c^*	*95% CI*	*p-het*
**Smoking status** (1 year before the interview)																																	
Never smoker	1142	638	1.00		173	1.00		81	1.00			237	155	1.00		46	1.00		19	1.00			904	483	1.00		127	1.00		62	1.00		
Former smoker ≥10	180	122	1.05	0.79–1.41	20	0.54	0.30–0.97	9	0.49	0.21–1.17	0.028	53	44	1.42	0.86–2.34	9	1.18	0.51–2.73	2	0.75	0.16–3.53	0.680	126	78	0.84	0.56–1.25	11	0.28	0.11–0.68	7	0.42	0.12–1.47	0.040
Former smoker <10	189	109	0.91	0.67–1.24	35	1.04	0.65–1.65	11	0.43	0.18–1.05	0.207	80	59	1.21	0.78–1.88	15	1.08	0.53–2.24	5	0.62	0.17–2.26	0.596	109	50	0.71	0.43–1.15	20	0.96	0.48–1.93	6	0.42	0.09–1.93	0.531
Active smoker	390	267	1.05	0.83–1.32	71	1.00	0.69–1.44	33	0.83	0.49–1.42	0.703	175	142	1.37	0.97–1.93	35	1.26	0.72–2.19	23	1.51	0.69–3.29	0.917	213	124	0.91	0.64–1.29	36	0.64	0.35–1.15	10	0.57	0.22–1.52	0.387
p-trend				0.850			0.936			0.287					0.089			0.447			0.383					0.392			0.167			0.170	
**Age at smoking initiation** (years)																																	
Never smoker	1142	638	1.00		173	1.00		81	1.00			237	155	1.00		46	1.00		19	1.00			904	483	1.00		127	1.00		62	1.00		
≥18	369	244	1.02	0.82–1.28	55	0.82	0.56–1.20	26	0.65	0.36–1.17	0.216	101	96	1.61	1.09–2.39	20	1.35	0.72–2.54	10	1.14	0.42–3.12	0.719	267	148	0.82	0.60–1.12	35	0.55	0.32–0.94	16	0.52	0.21–1.25	0.144
<18	389	258	1.01	0.80–1.28	72	0.96	0.66–1.10	27	0.65	0.37–1.16	0.330	209	153	1.22	0.87–1.70	39	1.11	0.65–1.92	20	1.14	0.51–2.51	0.942	178	104	0.89	0.60–1.32	33	0.65	0.34–1.23	7	0.45	0.14–1.43	0.176
p-trend				0.890			0.717			0.102					0.222			0.667			0.745					0.213			0.030			0.144	
**Duration of tobacco use** (years)																																	
Never smoker	1142	638	1.00		173	1.00		81	1.00			237	155	1.00		46	1.00		19	1.00			904	483	1.00		127	1.00		62	1.00		
≤20	229	163	1.14	0.87–1.50	36	0.84	0.53–1.34	22	0.78	0.40–1.51	0.281	130	100	1.46	1.00–2.14	23	1.15	0.62–2.11	13	1.03	0.41–2.59	0.607	98	63	0.89	0.57–1.38	13	0.39	0.16–0.94	9	0.51	0.15–1.82	0.162
20–30	228	162	1.02	0.77–1.33	50	1.10	0.72–1.67	18	0.71	0.37–1.37	0.511	138	102	1.11	0.76–1.61	27	1.10	0.60–2.03	12	1.04	0.47–2.65	0.991	89	60	1.04	0.65–1.67	23	1.04	0.52–2.10	6	0.67	0.18–2.45	0.804
>30	299	170	0.91	0.71–1.18	40	0.77	0.50–1.19	13	0.52	0.25–1.05	0.255	40	42	1.75	1.00–3.07	9	1.81	0.73–4.47	5	2.37	0.64–8.86	0.903	258	127	0.75	0.53–1.06	31	0.52	0.29–0.93	8	0.42	0.15–1.16	0.301
p-trend				0.561			0.418			0.048					0.109			0.332			0.446					0.141			0.048			0.087	
**Intensity** (cigarettes/day)																																	
Never smoker	1142	638	1.00		173	1.00		81	1.00			237	155	1.00		46	1.00		19	1.00			904	483	1.00		127	1.00		62	1.00		
<15	363	238	1.03	0.82–1.30	57	0.87	0.59–1.27	24	0.57	0.31–1.04	0.132	153	125	1.35	0.95–1.91	29	1.11	0.63–1.98	11	0.84	0.34–2.07	0.524	209	112	0.80	0.56–1.14	28	0.53	0.29–0.97	13	0.28	0.08–0.97	0.135
≥15	371	251	1.02	0.81–1.29	67	0.92	0.63–1.34	29	0.78	0.45–1.36	0.589	149	118	1.40	0.98–2.01	30	1.38	0.78–2.46	19	1.61	0.70–3.73	0.945	220	133	0.89	0.63–1.26	37	0.62	0.34–1.12	10	0.77	0.32–1.87	0.494
p-trend				0.827			0.600			0.249					0.058			0.273			0.316					0.395			0.054			0.320	
**Cumulative exposure** (pack-years)																																	
Never smoker	1142	638	1.00		173	1.00		81	1.00			237	155	1.00		46	1.00		19	1.00			904	483	1.00		127	1.00		62	1.00		
<10	256	168	1.00	0.77–1.30	39	0.83	0.53–1.28	19	0.60	0.31–1.17	0.272	127	94	1.32	0.91–1.92	21	0.99	0.83–1.84	10	0.86	0.33–2.23	0.506	128	73	0.75	0.49–1.14	18	0.50	0.24–1.04	9	0.40	0.11–1.39	0.405
10–25	240	149	0.93	0.71–1.22	41	0.93	0.60–1.43	19	0.70	0.36–1.37	0.716	105	93	1.42	0.96–2.11	24	1.51	0.82–2.80	10	1.28	0.48–3.37	0.953	134	56	0.71	0.45–1.10	17	0.43	0.19–0.96	9	0.51	0.14–1.78	0.475
>25	234	164	1.11	0.85–1.46	43	0.94	0.60–1.47	15	0.74	0.38–1.44	0.408	68	51	1.31	0.81–2.12	14	1.38	0.63–3.00	10	1.83	0.62–5.43	0.835	165	113	1.06	0.73–1.54	29	0.77	0.42–1.44	5	0.66	0.23–1.87	0.458
p-trend				0.662			0.712			0.231					0.099			0.211			0.295					0.771			0.133			0.256	
**Years smoking before first birth^a^**																																	
Never smoker	939	526	1.00		147	1.00		66	1.00			162	114	1.00		36	1.00		14	1.00			776	412	1.00		111	1.00		52	1.00		
<10	253	170	1.02	0.77–1.35	40	0.69	0.43–1.11	20	0.66	0.33–1.32	0.171	87	78	1.43	0.90–2.28	19	1.05	0.49–2.23	10	1.17	0.44–3.44	0.698	165	92	0.79	0.52–1.19	21	0.34	0.16–0.75	10	0.35	0.09–1.32	0.075
≥10	260	174	1.03	0.77–1.38	38	0.82	0.50–1.34	18	0.67	0.33–1.33	0.369	132	106	1.22	0.80–1.88	20	0.93	0.44–1.96	12	1.18	0.42–3.32	0.773	127	68	0.99	0.62–1.58	18	0.67	0.31–1.48	6	0.52	0.13–1.99	0.449
p-trend				0.838			0.243			0.178					0.264			0.854			0.730					0.713			0.068			0.172	
**Cigarettes per day before first birth^a^**																																	
Never smoker	939	526	1.00		147	1.00		66	1.00			162	114	1.00		36	1.00		14	1.00			776	412	1.00		111	1.00		52	1.00		
<15	226	146	1.01	0.76–1.35	28	0.58	0.34–0.99	18	0.67	0.33–1.37	0.092	95	85	1.43	0.92–2.21	17	0.91	0.42–1.94	6	0.86	0.27–2.71	0.395	130	61	0.73	0.47–1.16	11	0.27	0.10–0.72	12	0.30	0.07–1.39	0.097
≥15	212	139	0.97	0.72–1.31	36	0.94	0.58–1.52	12	0.62	0.29–1.32	0.517	88	74	1.29	0.81–2.03	17	1.37	0.66–2.85	8	1.10	0.35–3.46	0.945	123	65	0.90	0.56–1.44	19	0.72	0.34–1.55	4	0.73	0.21–2.56	0.836
p-trend				0.878			0.491			0.163					0.229			0.464			0.925					0.442			0.117			0.368	

RRR: relative risk ratio. Multinomial logistic regression analyses were used to test whether each tobacco variable was associated with specific tumor subtypes. The p-trend values were calculated by incorporating the categorized variable as a continuous variable in the multivariable models. The difference of effects of the corresponding variable between tumor subtypes was calculated as p-value of heterogeneity.

a Only parous women.

b RRR and 95% CI adjusted for age, education level, region, BMI, age at first birth, number of children, previous biopsies, family history of breast cancer, menopausal status and alcohol use.

c RRR and 95% CI adjusted for age, education level, region, age at first birth, number of children, previous biopsies, family history of breast cancer and alcohol and hormonal contraceptive use.

d RRR and 95% CI adjusted for age, education level, region, BMI, age at first birth, number of children, previous biopsies, family history of breast cancer, history of hormone replacement therapy and age at menopause. p-het: heterogeneity of effects of each tobacco variable between tumor subtypes.

## DISCUSSION

Our results suggest that the association of cigarette smoking and breast cancer risk is modulated by menopausal status. Thus, we observed an increase of risk limited to pre-menopausal women, which was more apparent among long-term smokers, among women who started smoking after the age of 18 years, and for HR+ tumor subtype. In contrast, post-menopausal women who smoked had lower risk of breast cancer than non-smokers, especially among long-term smokers and for HER2+ tumor subtype; this decrease was, however, restricted to overweight and obese women.

These findings are partly in accordance with our expectations. Over recent decades, evidence surrounding the role of tobacco in breast cancer has been inconsistent and inconclusive. However, in the past years, large sample cohort studies and meta analyses have consistently reported a small increase in the risk of breast cancer associated with cigarette smoking, in agreement with our results^2,5,18-22^. Similarly, several studies where analyses were conducted separately according to menopausal status, frequently reported no risk increase for breast cancer, or even protective effects, among post-menopausal women with a smoking history^14,23,24^, although some recent cohorts with a large number of incident cases found an increase in breast cancer risk among both pre- and post-menopausal women^2,5,19,21,22,25^. The increase, nevertheless, has been consistently reported to be greater before menopause^2,5,19,22^. We cannot rule out that the discrepancies with our results might be due to residual confounding, or related to the low number of cases in certain subgroup analyses; another possible explanation may lie in the differences in tobacco use among the studied population, as the prevalence, duration and intensity of smoking have been historically higher in American and Nordic cohorts^2,21,22,25^, compared to Spanish cohorts – especially older women^26^.

The combined effects of obesity and tobacco smoking, on the other hand, have received less attention, but our findings are concordant with previous studies^12^. In contrast, we have not found any risk increase among women who smoked heavily before their first full-term pregnancy, even though this association has been consistently described in the literature^2,4,5,18,20-22^.

According to our results, menopause plays a relevant role in the relationship between breast cancer risk and tobacco exposure. Tobacco has both carcinogenic and antiestrogenic activity. During pre-menopause, the carcinogenic stimulus of tobacco may outweigh its antiestrogenic effects, which can be insufficient to counteract the high estrogen levels, altogether increasing the risk of breast cancer. After menopause, in spite of tobacco retaining its carcinogenic potential, mammary cells become less susceptible to stimuli, and estrogen levels are further reduced due to the antiestrogenic activity of tobacco, as nicotine and other tobacco compounds interfere with aromatase, a key enzyme in the metabolic pathway of estrone^11^. The joint outcome might present itself either as a balanced risk between carcinogenic stimulus and hormonal blockade, or as a risk decrease due to a higher relevance of antiestrogenic effects^24^. This ‘dual’ effect of tobacco may explain our different results among pre- and post-menopausal women, as well as the lack of a consistent association between smoking and breast cancer reported by some studies, especially those which did not assess pre- and post-menopausal women separately^27,28^. The seemingly opposite effects of smoking according to menopausal status reported here have been also found in other studies^18,23,24,29^. In the EPIC Study, a large European cohort including 25343 Spanish women^29^, the authors found an increase of risk in women smoking before menopause, reporting a dose-response effect for cigarette consumption between menarche and first birth; however, after menopause, an increase of pack-years was associated with a decrease in risk. A study by van den Brandt^24^ found the highest breast cancer risk among women smoking a high number of pack-years before menopause and no cigarette consumption afterwards; in contrast, the risk was significantly decreased among women who were light smokers before menopause and became heavy smokers thereafter.

Another interesting result of our study, also related to this dual effect, is the possible role of obesity as modulator of the relationship between tobacco use and breast cancer among post-menopausal women. Our data hint at differences in breast cancer risk according to BMI. Obesity has been recognized as a risk factor for post-menopausal breast cancer, but not for pre-menopausal cases^13,30-32^. Before menopause, the ovaries are the main source of estrogens. After ovarian decline and menopause, adipose tissue becomes the main producer of estrogens in the form of estrone. While pre-menopausal obesity has little to no effect in contributing to the high levels of estrogens produced by the ovaries, high amounts of adipose tissue after menopause will significantly increase those levels, stimulating the growth of estrogen-sensitive breast tumours^13^. Similarly, this dual effect might help to explain why, in our data, the highest risk reduction among post-menopausal women is not found among heavy smokers, but rather among those with a moderate tobacco use, especially women exposed to a low-moderate intensity sustained over a long period of time. Perhaps, above certain doses, the carcinogenic effects might exceed the antiestrogenic effects of tobacco even in this subgroup of women.

Regarding the relationship with tobacco by tumor types, we have not found differences among them in pre-menopausal cancers, probably due to the low number of cases of HER2+ and TN tumors, as the HR+ subtype constitutes >70% of all tumors in this group. On the other hand, for post-menopausal women, TN seemed to have the lowest risks, although in most cases the tests for heterogeneity suggest that there might not be differences by subgroup. In the literature, several studies have reported a stronger association between cigarette smoking and ER+ breast cancer. Without considering differences by menopausal status, a pooled analysis of 14 cohort studies of nearly one million participants^20^ reported an increased risk among smokers for both, ER+ and ER- breast cancer, yet the effect was stronger and more consistent for ER+ tumors. Also, in the EPIC Study^29^, smoking appeared to be a risk factor for ER+, but not for ER- tumors (including ER-/PR+), although the authors, again, did not provide risk estimators by menopausal status. Nevertheless, there are a few studies that have evaluated pre-menopausal cases – a cohort^33^ and a population-based case-control^34^ study which reported an increased risk of breast cancer with smoking in ER+ –, or, in post-menopausal women, two case-control studies^7,35^ which found no association between smoking and ER+ breast cancer, and a Norwegian nested case-control^15^, including women aged 50–69 years, that found an increase of risk for ER+ and ER-/PR+ tumors. To our knowledge, no previous studies have reported an association between decreased post-menopausal HER2+ breast cancer risk and cigarette smoking. However, researchers studying subtypes usually focus on estrogen and progesterone receptor status to establish tumor subtype; it is also relatively usual to combine pre- and post-menopausal women^7,19,20,29,33-35^. Therefore, any specific association could have been overlooked, especially due to the low frequency of HER2+ tumors, compared to ER+ tumors. Only one nested case-control study^15^ researching the impact of alcohol, physical activity and smoking explicitly reported no association between these factors and HER2+ tumors.

### Strengths and limitations

Our study has several strengths. The comprehensive evaluation of smoking history has enabled us to classify participants according to smoking status, age at initiation, duration and intensity, both overall and in relation to reproductive milestones, and to provide a global view of the relationship between breast cancer and tobacco use. Also, the MCC-Spain study, which is the largest case-control cancer study done in Spanish population, has the advantage of counting with randomly selected general population controls, as well as with incident cases. This last factor reduces the risk of survivorship bias, which could be a problem if tobacco smoking played any role in tumor progression, with higher rates of breast cancer proliferation, recurrence, and mortality^3^. The wide age range of participants allowed the separate study of pre- and post-menopausal women, and the availability of clinical data allowed the possibility of evaluating the relationship between cigarette smoking and specific tumor subtypes by menopausal status. Also, we have explored the possible role of BMI as modulating factor in postmenopausal women, adding new information to this complex picture.

On the other hand, our work presents certain limitations. First, as stated above, smoking prevalence was low among older women, which, combined with the low number of cases in certain subgroups, limited our capacity to explore the association among them. Thus, we cannot rule out the possibility that our findings may be partly due to chance, and that they might not reflect any actual effects of tobacco use on breast cancer risk. However, current evidence suggests otherwise, and, in our study, both the risk increase in pre-menopausal and the decrease in post-menopausal women were consistent across smoking variables. Likewise, even though we tried to address the risk of confounding with our comprehensive inclusion of well-established risk factors of breast cancer, they may be still residual confounding. Second, given our case-control design, the risk of having certain recall bias is unavoidable, and we cannot know whether it is differential or non-differential. Nevertheless, we tried to minimize this risk by administering a common and comprehensive questionnaire about tobacco use by a trained interviewer. Also, most women do not consider tobacco as risk factor for breast cancer, which would favor a non-differential bias. Third, response bias was a concern for our study, as women with a higher education level seemed more prone to participate. In this sense, the pattern of female tobacco use in Spain has evolved: among older women, smoking was uncommon except for university-educated women; during the last decades, smoking has become widespread in society and is currently more frequent among women of low economic and education level^26^. To prevent a possible confounding effect, education level was included as an adjusting variable in all our analyses. Lastly, the generalizability of our findings might be limited, as our study was conducted within the Spanish population, which may differ from other countries in both genetic traits and lifestyle habits.

## CONCLUSIONS

The role of tobacco as a breast cancer risk factor appears to be modified by menopausal status and obesity. The effect of cigarette smoke might depend on breast differentiation and estrogen metabolism in each specific life period. In our study, pre-menopausal women show a modest increase of breast cancer risk, suggesting that the carcinogenic properties of tobacco prevail among young women, whereas the decreased risk among post-menopausal women is consistent with the antiestrogenic effects of tobacco. The harmful effects of tobacco on other organs and its role as a risk factor for other types of cancer, however, largely exceed this observed protective influence. Therefore, our findings support the development of early smoking prevention programs.

## Data Availability

The datasets used for the current study are not publicly available due to restrictions in the informed consent, but are available from the corresponding author on reasonable request.
