# Supplementary material for: Smoking history and breast cancer risk by pathological subtype: MCC-Spain study
Source: Tob Induc Dis. 2023 Nov 30;21:157. doi: 10.18332/tid/174132 (PMC10688255; doi:10.18332/tid/174132)
Supplement: Supplementary file 1 [file TID-21-157-s1.pdf]

Supplementary Table S1 Association between cigarette smoking variables and breast cancer risk, overall and by menopausal status, additionally adjusted by diet-related variables. Multi case-control study (MCC-Spain), 1733 breast cancer cases + 1903 controls, 2008-2013, Spain.

Smoking variables	All Women (N = 3636; Cases = 1733; Controls = 1903)						Premenopausal Women (N = 1157; Cases = 610; Controls = 547)						Postmenopausal Women (N = 2474; Cases = 1122; Controls = 1352)						P-int [f]
	Ca	Co	OR[b]	95% CI	OR[c]	95% CI	Ca	Co	OR[b]	95% CI	OR[d]	95% CI	Ca	Co	OR[b]	95% CI	OR[e]	95% CI	
**Smoking status (1 year before the interview)**																			
Never smoker	972	1142	1.00		1.00		242	237	1.00		1.00		730	904	1.00		1.00		
Former smoker ≥10	170	180	1.12	0.88-1.41	1.02	0.77-1.36	61	53	1.16	0.76-1.77	1.46	0.87-2.46	109	126	1.01	0.76-1.35	0.81	0.54-1.22	
Former smoker <10	168	189	0.90	0.71-1.15	0.85	0.63-1.16	83	80	1.01	0.70-1.45	0.94	0.59-1.51	85	109	0.78	0.57-1.07	0.70	0.42-1.14	
Active smoker	413	390	1.03	0.87-1.24	1.04	0.82-1.32	219	175	1.19	0.91-1.57	1.29	0.88-1.88	193	213	0.87	0.68-1.11	0.81	0.56-1.17	
p-trend				*0.892*		*0.940*				*0.259*		*0.294*				*0.162*		*0.162*	*0.214*
**Age at smoking initiation**																			
Never smoker	972	1142	1.00		1.00		242	237	1.00		1.00		730	904	1.00		1.00		
≥18	367	369	1.09	0.91-1.30	1.01	0.80-1.27	140	101	1.33	0.97-1.83	1.49	0.98-2.26	227	267	0.93	0.75-1.16	0.73	0.53-1.01	
<18	389	389	0.97	0.81-1.17	0.98	0.77-1.24	227	209	1.05	0.80-1.37	1.11	0.77-1.58	161	178	0.85	0.65-1.11	0.92	0.62-1.38	
p-trend				*0.955*		*0.886*				*0.686*		*0.576*				*0.219*		*0.362*	*0.036*
**Duration of tobacco use (years)**																			
Never smoker	972	1142	1.00		1.00		242	237	1.00		1.00		730	904	1.00		1.00		
≤20	247	229	1.08	0.87-1.34	1.07	0.82-1.41	151	130	1.17	0.87-1.59	1.39	0.93-2.08	96	98	1.05	0.76-1.44	0.85	0.54-1.34	
20-30	249	228	1.07	0.86-1.32	1.02	0.78-1.34	150	138	1.01	0.75-1.37	0.95	0.63-1.43	99	89	1.09	0.78-1.51	1.01	0.62-1.62	
>30	251	299	0.96	0.79-1.16	0.89	0.69-1.16	61	40	1.45	0.91-2.30	1.60	0.89-2.87	189	258	0.77	0.61-0.97	0.69	0.48-0.98	
p-trend				*0.863*		*0.504*				*0.284*		*0.353*				*0.052*		*0.058*	*0.012*
**Intensity (no. of cigarettes per day)**																			
Never smoker	972	1142	1.00		1.00		242	237	1.00		1.00		730	904	1.00		1.00		
<15	356	363	1.00	0.84-1.20	0.98	0.78-1.23	180	153	1.13	0.85-1.51	1.26	0.87-1.82	175	209	0.85	0.67-1.09	0.70	0.49-1.01	
≥15	384	371	1.09	0.91-1.30	1.04	0.82-1.31	180	149	1.18	0.88-1.58	1.25	0.84-1.88	204	220	0.97	0.77-1.23	0.90	0.63-1.30	
p-trend				*0.378*		*0.815*				*0.244*		*0.232*				*0.655*		*0.375*	*0.105*
**Cumulative exposure (pack-years)**																			
Never smoker	972	1142	1.00		1.00		242	237	1.00		1.00		730	904	1.00		1.00		
<10	254	256	0.98	0.80-1.21	1.01	0.78-1.31	140	127	1.08	0.79-1.47	1.35	0.91-1.99	113	128	0.89	0.66-1.19	0.73	0.48-1.12	
10-25	228	240	0.97	0.78-1.20	0.86	0.66-1.14	133	105	1.23	0.90-1.69	1.11	0.72-1.71	95	134	0.74	0.55-1.00	0.61	0.39-0.96	
>25	247	234	1.16	0.94-1.42	1.12	0.85-1.47	82	68	1.15	0.78-1.68	1.23	0.73-2.07	165	165	1.05	0.81-1.36	1.03	0.70-1.52	
p-trend				*0.297*		*0.816*				*0.248*		*0.427*				*0.733*		*0.548*	*0.131*
**Years smoking before first birth [a]**																			
Never smoker	801	939	1.00		1.00		177	162	1.00		1.00		624	776	1.00		1.00		
<10	255	253	0.99	0.80-1.23	0.96	0.72-1.27	118	87	1.18	0.82-1.70	1.22	0.73-2.03	137	165	0.85	0.64-1.12	0.73	0.48-1.12	
≥10	253	260	0.97	0.78-1.21	0.97	0.73-1.30	148	132	1.06	0.77-1.47	1.08	0.68-1.71	105	127	0.87	0.63-1.19	0.97	0.60-1.56	
p-trend				*0.789*		*0.805*				*0.686*		*0.674*				*0.267*		*0.580*	*0.255*
**Cigarettes per day before first birth [a]**																			
Never smoker	801	939	1.00		1.00		177	162	1.00		1.00		624	776	1.00		1.00		
<15	211	226	0.92	0.73-1.16	0.93	0.70-1.25	117	95	1.09	0.77-1.56	1.22	0.76-1.95	94	130	0.77	0.56-1.05	0.68	0.43-1.07	
≥15	206	212	0.98	0.78-1.24	0.97	0.71-1.31	106	88	1.15	0.80-1.66	1.05	0.62-1.78	100	123	0.86	0.63-1.18	0.99	0.61-1.59	
p-trend				*0.775*		*0.759*				*0.441*		*0.759*				*0.208*		*0.604*	*0.199*

[i] OR: odds ratio. Multivariable logistic regression analyses were used to test whether each tobacco variable was associated with breast cancer risk. The p-trend values were calculated by incorporating the categorised variable as a continuous variable in the multivariable models. The p-value of the interaction term between menopausal status and the corresponding variable was calculated using the likelihood ratio test. [a] Only parous women. [b] OR and 95% CI adjusted for age, study level and region. [c] OR and 95% CI adjusted for age, study level, region, BMI, age at first birth, number of children, previous biopsies, family history of breast cancer, menopausal status, alcohol use, dietary pattern, daily caloric intake and antioxidant activity. [d] OR and 95% CI adjusted for age, study level, region, age at first birth, number of children, previous biopsies, family history of breast cancer, hormonal contraceptive use, alcohol use, dietary pattern, daily caloric intake and antioxidant activity. [e] OR and 95% CI adjusted for age, study level, region, BMI, age at first birth, number of children, previous biopsies, family history of breast cancer, history of hormone replacement therapy, age at menopause, alcohol use, dietary pattern, daily caloric intake and antioxidant activity. [f] P-int.: *P* value of the interaction term.

Supplementary Table S2 Association between cigarette smoking variables and breast cancer subtypes, overall and by menopausal status, additionally adjusted by diet-related variables.
Multi case-control study (MCC-Spain), 1733 breast cancer cases + 1903 controls, 2008-2013, Spain.

Smoking variables	**All Women** (N = 3636; Cases = 1733; Controls = 1903)											**Premenopausal Women** (N = 1157; Cases = 610; Controls = 547)											**Postmenopausal Women** (N = 2474; Cases = 1122; Controls = 1352)										
	Co	HR+ (N = 1144)			HER2+ (N = 300)			TN (N = 134)				Co	HR+ (N = 405)			HER2+ (N = 105)			TN (N = 49)				Co	HR+ (N = 738)			HER2+ (N = 195)			TN (N = 85)			
	n	n	RRR[a]	95% CI	n	RRR[a]	95% CI	n	RRR[a]	95% CI	p-het	n	n	RRR[b]	95% CI	n	RRR[b]	95% CI	n	RRR[b]	95% CI	p-het	n	n	RRR[c]	95% CI	n	RRR[c]	95% CI	n	RRR[c]	95% CI	p-het
**Smoking status** (1 year before the interview)																																	
Never smoker	1142	638	1.00		173	1.00		81	1.00			237	155	1.00		46	1.00		19	1.00			904	483	1.00		127	1.00		62	1.00		
Former smoker ≥10	180	122	1.12	0.81-1.55	20	0.61	0.31-1.19	9	0.59	0.22-1.53	0.120	53	44	1.51	0.85-2.68	9	1.42	0.45-4.45	2	1.32	0.25-7.06	0.985	126	78	0.96	0.61-1.52	11	0.38	0.15-0.95	7	0.40	0.09-1.89	0.106
Former smoker <10	189	109	0.90	0.64-1.26	35	1.10	0.63-1.92	11	0.39	0.13-1.13	0.217	80	59	1.09	0.65-1.81	15	1.41	0.54-3.65	5	0.21	0.03-1.77	0.268	109	50	0.68	0.38-1.22	20	0.99	0.42-2.31	6	0.62	0.12-3.23	0.716
Active smoker	390	267	1.08	0.83-1.40	71	0.98	0.62-1.55	33	0.84	0.45-1.57	0.717	175	142	1.30	0.85-1.97	35	1.35	0.63-2.90	23	1.48	0.56-3.94	0.963	213	124	0.88	0.58-1.34	36	0.68	0.34-1.37	10	0.42	0.11-1.56	0.471
p-trend				*0.744*			*0.990*			*0.356*					*0.295*			*0.428*			*0.699*					*0.402*			*0.306*			*0.163*	
**Age at smoking initiation**																																	
Never smoker	1142	638	1.00		173	1.00		81	1.00			237	155	1.00		46	1.00		19	1.00			904	483	1.00		127	1.00		62	1.00		
≥18	369	244	1.06	0.82-1.38	55	0.85	0.53-1.35	26	0.64	0.31-1.29	0.283	101	96	1.52	0.95-2.42	20	1.71	0.73-4.03	10	0.83	0.23-2.93	0.607	267	148	0.82	0.57-1.19	35	0.52	0.27-0.99	16	0.44	0.14-1.43	0.283
<18	389	258	1.03	0.79-1.35	72	0.97	0.61-1.53	27	0.69	0.36-1.33	0.501	209	153	1.18	0.80-1.75	39	1.22	0.58-2.55	20	1.12	0.43-2.96	0.991	178	104	1.00	0.63-1.58	33	0.87	0.42-1.78	7	0.47	0.12-1.86	0.559
p-trend				*0.775*			*0.806*			*0.207*					*0.418*			*0.591*			*0.840*					*0.746*			*0.342*			*0.164*	
**Duration of tobacco use** (years)																																	
Never smoker	1142	638	1.00		173	1.00		81	1.00			237	155	1.00		46	1.00		19	1.00			904	483	1.00		127	1.00		62	1.00		
≤20	229	163	1.13	0.83-1.54	36	0.84	0.48-1.49	22	0.88	0.41-1.88	0.533	130	100	1.43	0.92-2.23	23	1.59	0.70-3.60	13	0.92	0.30-2.83	0.715	98	63	0.94	0.56-1.58	13	0.43	0.16-1.17	9	0.86	0.22-3.34	0.339
20-30	228	162	1.08	0.79-1.46	50	1.01	0.60-1.72	18	0.71	0.33-1.52	0.577	138	102	1.02	0.65-1.59	27	1.07	0.46-2.52	12	0.91	0.29-2.84	0.972	89	60	1.25	0.74-2.12	23	0.90	0.38-2.12	6	0.25	0.03-2.09	0.279
>30	299	170	0.94	0.70-1.26	40	0.87	0.53-1.44	13	0.48	0.20-1.12	0.309	40	42	1.66	0.88-3.12	9	1.71	0.54-5.37	5	2.26	0.42-11.99	0.938	258	127	0.71	0.47-1.07	31	0.64	0.33-1.23	8	0.37	0.10-1.40	0.626
p-trend				*0.839*			*0.676*			*0.072*					*0.276*			*0.465*			*0.698*					*0.202*			*0.199*			*0.082*	
**Intensity** (no. of cigarettes per day)																																	
Never smoker	1142	638	1.00		173	1.00		81	1.00			237	155	1.00		46	1.00		19	1.00			904	483	1.00		127	1.00		62	1.00		
<15	363	238	1.04	0.80-1.35	57	0.89	0.56-1.41	24	0.61	0.31-1.21	0.287	153	125	1.31	0.87-1.97	29	1.44	0.68-3.02	11	0.94	0.34-2.62	0.782	209	112	0.84	0.56-1.26	28	0.51	0.25-1.03	13	0.13	0.02-1.01	0.103
≥15	371	251	1.06	0.81-1.39	67	0.94	0.59-1.51	29	0.78	0.40-1.52	0.636	149	118	1.30	0.84-2.04	30	1.42	0.61-3.32	19	1.17	0.39-3.52	0.960	220	133	0.92	0.61-1.39	37	0.76	0.38-1.51	10	0.94	0.31-2.81	0.876
p-trend				*0.653*			*0.749*			*0.329*					*0.206*			*0.363*			*0.817*					*0.584*			*0.253*			*0.503*	
**Cumulative exposure** (pack-years)																																	
Never smoker	1142	638	1.00		173	1.00		81	1.00			237	155	1.00		46	1.00		19	1.00			904	483	1.00		127	1.00		62	1.00		
<10	256	168	1.11	0.83-1.49	39	0.75	0.44-1.30	19	0.62	0.29-1.35	0.173	127	94	1.45	0.95-2.23	21	1.18	0.51-2.73	10	0.91	0.30-2.73	0.662	128	73	0.87	0.54-1.40	18	0.47	0.20-1.12	9	0.20	0.02-1.57	0.184
10-25	240	149	0.85	0.62-1.17	41	1.04	0.62-1.73	19	0.63	0.28-1.42	0.557	105	93	1.11	0.69-1.79	24	2.05	0.91-4.59	10	0.89	0.24-3.24	0.317	134	56	0.71	0.42-1.19	17	0.43	0.17-1.09	9	0.42	0.09-1.99	0.535
>25	234	164	1.15	0.85-1.58	43	0.99	0.57-1.73	15	0.85	0.39-1.85	0.692	68	51	1.24	0.70-2.20	14	0.90	0.26-3.10	10	1.65	0.44-6.24	0.790	165	113	1.04	0.66-1.62	29	1.01	0.49-2.07	5	0.86	0.24-3.05	0.961
p-trend				*0.731*			*0.972*			*0.400*					*0.484*			*0.398*			*0.623*					*0.743*			*0.489*			*0.467*	
**Years smoking before first birth** [a]																																	
Never smoker	939	526	1.00		147	1.00		66	1.00			162	114	1.00		36	1.00		14	1.00			776	412	1.00		111	1.00		52	1.00		
<10	253	170	1.00	0.72-1.37	40	0.88	0.51-1.55	20	0.68	0.32-1.47	0.622	87	78	1.21	0.69-2.12	19	1.46	0.50-4.24	10	0.76	0.20-2.84	0.728	165	92	0.87	0.54-1.40	21	0.46	0.19-1.09	10	0.65	0.15-2.75	0.382
≥10	260	174	1.08	0.78-1.50	38	0.74	0.40-1.40	18	0.57	0.25-1.31	0.213	132	106	1.22	0.74-2.02	20	0.82	0.27-2.54	12	0.84	0.24-2.88	0.694	127	68	1.16	0.68-1.97	18	0.87	0.35-2.14	6	0.24	0.03-2.09	0.333
p-trend				*0.676*			*0.349*			*0.141*					*0.411*			*0.890*			*0.723*					*0.766*			*0.385*			*0.167*	
**Cigarettes per day before first birth** [a]																																	
Never smoker	939	526	1.00		147	1.00		66	1.00			162	114	1.00		36	1.00		14	1.00			776	412	1.00		111	1.00		52	1.00		
<15	226	146	1.02	0.74-1.41	28	0.68	0.36-1.27	18	0.71	0.32-1.58	0.369	95	85	1.28	0.77-2.14	17	1.38	0.48-3.96	6	0.74	0.19-2.89	0.721	130	61	0.87	0.52-1.45	11	0.36	0.13-0.99	12	0.19	0.02-1.61	0.121
≥15	212	139	0.99	0.70-1.41	36	0.97	0.53-1.77	12	0.49	0.19-1.28	0.364	88	74	1.09	0.61-1.94	17	1.44	0.47-4.45	8	0.31	0.05-1.76	0.309	123	65	1.03	0.60-1.79	19	0.85	0.34-2.12	4	1.06	0.24-4.78	0.919
p-trend				*0.997*			*0.693*			*0.121*					*0.684*			*0.488*			*0.188*					*0.974*			*0.345*			*0.610*	

[i] RRR: relative risk ratio. Multinomial logistic regression analyses were used to test whether each tobacco variable was associated with specific tumour subtypes. The p-trend values were calculated by incorporating the categorised variable as a continuous variable in the multivariable models. The difference of effects of the corresponding variable between tumour subtypes was calculated as p-value of heterogeinity. [a] Only parous women. [b] RRR and 95% CI adjusted for age, study level, region, BMI, age at first birth, number of children, previous biopsies, family history of breast cancer, menopausal status, alcohol use, dietary pattern, daily caloric intake and antioxidant activity. [c] RRR and 95% CI adjusted for age, study level, region, age at first birth, number of children, previous biopsies, family history of breast cancer, hormonal contraceptive use, alcohol use, dietary pattern, daily caloric intake and antioxidant activity. [d] RRR and 95% CI adjusted for age, study level, region, BMI, age at first birth, number of children, previous biopsies, family history of breast cancer, history of hormone replacement therapy, age at menopause, alcohol use, dietary pattern, daily caloric intake and antioxidant activity.

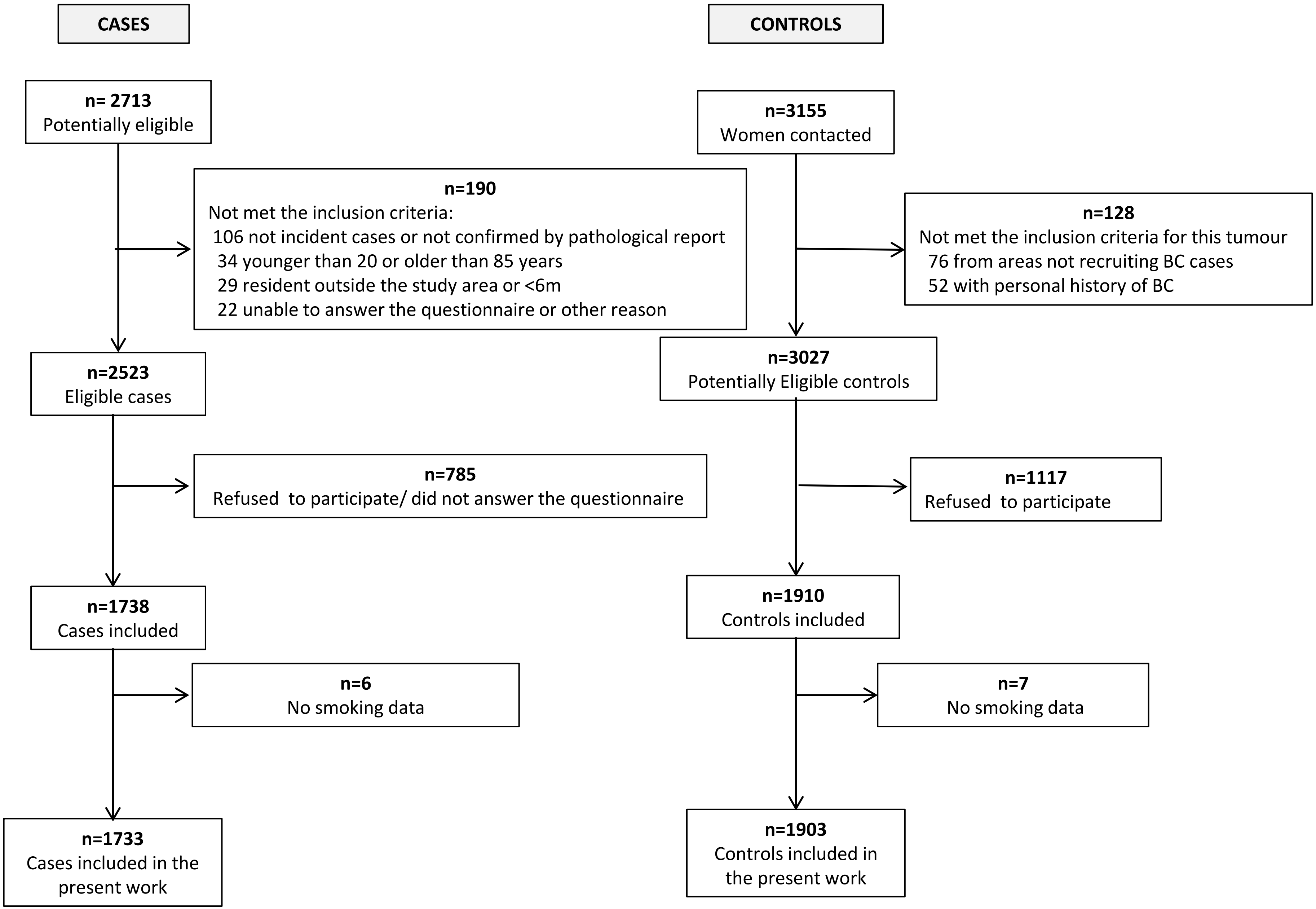
CASES
CONTROLS
n= 2713
Potentially eligible
n=3155
Women contacted
n=190
Not met the inclusion criteria:
106 not incident cases or not confirmed by pathological report
34 younger than 20 or older than 85 years
29 resident outside the study area or <6m
22 unable to answer the questionnaire or other reason
n=128
Not met the inclusion criteria for this tumour
76 from areas not recruiting BC cases
52 with personal history of BC
n=2523
Eligible cases
n=3027
Potentially Eligible controls
n=785
Refused to participate/ did not answer the questionnaire
n=1117
Refused to participate
n=1738
Cases included
n=1910
Controls included
n=6
No smoking data
n=7
No smoking data
n=1733
Cases included in the present work
n=1903
Controls included in the present work

Supplementary Fig. 1 Flowchart of response and selection of participants. Multi case-control study (MCC-Spain), 2008-2013, Spain.

© 2023 Peñalver-Argüeso B. et al.
